# Consensus of the Spanish society of laboratory medicine and the Spanish society of medical oncology on the methodology and criteria for evaluation of circulating tumour markers in breast cancer

**DOI:** 10.1007/s12094-020-02529-x

**Published:** 2021-02-07

**Authors:** F. Ayala de la Peña, B. Ortiz-Muñoz, T. Quintanar-Verdúguez, J. D. Santotoribio, S. de la Cruz, J. Trapé-Pujol, E. Galve-Calvo, J. M. Augé-Fradera, J. García-Gómez, Á. González-Hernández

**Affiliations:** 1 Department of Hematology and Medical Oncology, Sociedad Española de Oncología Médica (SEOM), Hospital General Universitario Morales Meseguer, Avda. Marqués de los Vélez, s/n, 30008 Murcia, Spain; 2 Service of Biochemistry, Sociedad Española de Medicina de Laboratorio (SEQCML), Fundación Instituto Valenciano de Oncología, Valencia, Spain; 3 Department of Medical Oncology, Sociedad Española de Oncología Médica (SEOM), Hospital General Universitario de Elche y Vega Baja, Elche, Spain; 4 Service of Biochemistry, Sociedad Española de Medicina de Laboratorio (SEQCML), Hospital Universitario Virgen del Rocío, Sevilla, Spain; 5 Department of Medical Oncology, Sociedad Española de Oncología Médica (SEOM), Complejo Hospitalario de Navarra, Pamplona, Spain; 6 Service of Biochemistry, Sociedad Española de Medicina de Laboratorio (SEQCML), Althaia Xarxa Assistencial Universitaria, Manresa, Spain; 7 Department of Medical Oncology, Sociedad Española de Oncología Médica (SEOM), Hospital Universitario de Basurto, Bilbao, Spain; 8 Service of Biochemistry, Sociedad Española de Medicina de Laboratorio (SEQCML), Hospital Clinic, Barcelona, Spain; 9 Department of Medical Oncology, Sociedad Española de Oncología Médica (SEOM), Complexo Hospitalario de Ourense, Ourense, Spain; 10 Service of Biochemistry, Sociedad Española de Medicina de Laboratorio (SEQCML), Clínica Universidad de Navarra, Pamplona, Spain

**Keywords:** Biomarkers, Breast neoplasms, Carcinoembryonic antigen, p53, Tumor suppressor

## Abstract

The measurement of circulating tumour markers (TMs) for the diagnosis or monitoring of breast cancer has sometimes been considered of limited utility. In addition to the overinterpretation of irrelevant changes in marker levels, the characteristics of the patient, the disease or other pathologies that can modify them are often not considered in their evaluation. On the other hand, there are recent data on the relationship of TMs with molecular subtypes and on their prognostic value, the knowledge of which may improve their clinical utility. This consensus article arises from a collaboration between the Spanish Society of Laboratory Medicine (SEQC^ML^) and the Spanish Society of Medical Oncology (SEOM). It aims to improve the use and interpretation of circulating TMs in breast cancer. The text summarizes the current knowledge and available evidence on the subject and proposes a series of recommendations mainly focussed on the indication, the frequency of testing and the factors that should be considered for correctly interpreting changes in the levels of TMs.

## Introduction

Breast cancer is the neoplasm with the highest incidence in women in Spain [1]. Although in recent years its prognosis has improved considerably and new treatments and diagnostic tests have been rapidly developed, the evaluation of circulating tumour markers (TMs) for the diagnosis or monitoring of this neoplasia is considered to have limited utility, to the point that they are included in the “do not use” recommendations [2, 3]. However, at variance with the recommendations of international guidelines that restrict their indication in the monitoring of metastatic disease, there is a high demand for the measurement of TMs in breast cancer in the current environment [4]. The evaluation of TM levels in this cancer is often inadequate, distinguishing only between elevated and normal marker levels without considering the minimum differences that may be relevant, the characteristics of the patient or the temporal evolution of the disease. In recent years, new circulating TMs have been discovered, and there is increasing evidence about how they relate to molecular subtypes and their prognostic value, which can increase their clinical utility [5].

In an environment in which better patient outcomes are achieved from multidisciplinary collaboration among professionals, the establishment of joint action guidelines by two scientific societies with different perspectives may facilitate adherence to scientific evidence-based recommendations, thereby improving the quality and efficiency of breast cancer care. With this in mind, the Spanish Society of Laboratory Medicine (SEQC^ML^) and the Spanish Society of Medical Oncology (SEOM) have formed a joint working group to develop recommendations that are applicable in today’s clinical environment and that will be useful for daily medical practice.

## Circulating tumour markers in breast cancer

### General considerations

A TM is considered to be any molecule produced by tumour cells, or by the body itself in response to a tumour, whose presence can be detected in serum or other biological fluids and which reflects tumour activity. Its quantification gives us insight into the presence, evolution, or therapeutic response of the tumour. Circulating TMs have multiple clinical applications: They can be used for screening and diagnosis, for tumour prognosis, and for assessing response to treatment, as well as for monitoring the course of the disease [6].

Both the sensitivity and specificity of a TM are influenced by factors specific to the tumour (histological type, degree of differentiation, stage and vascularization) or the TM itself (secretion, elimination and plasma half-life), and these parameters vary with the TMs used [7]. The correct interpretation of a TM is essential to avoid false positives and false negatives. Errors can occur in the different phases of the laboratory process (preanalytical, analytical, and postanalytical), which need to be identified to correctly interpret the results and assess their clinical impact [8].

Most errors in the preanalytical phase are due to the poor quality of the sample. In the analytical phase, the correct validation of the analytical method using regulatory agency protocols reduces the risk of errors. The most common errors are those due to cross-reactions with related molecules, interference from heterophilic antibodies, contamination between samples and the ‘prozone effect’. In the post-analytical phase, it is important to establish the reference values for the TM for each method used and to evaluate the concentrations in relation to the previous levels in each patient.

Methods for the detection of TM are not usually interchangeable, so it is advisable to use the same one during the monitoring of a patient; but if changing the TM measurement methodology is unavoidable during patient monitoring, it is important to take certain precautions. According to the recommendations of the National Academy of Clinical Biochemistry (NACB) [7], the new method must be validated beforehand, the physicians in charge should be informed of the change, and the TM should be measured for a period of at least 6 months with both analytical procedures.

TMs, like all biochemical magnitudes, present a within-subject coefficient of variation (CV_i_). In addition, all analytical procedures have an analytical coefficient of variation (CV_a_). Taking these into account, a change in the concentration of TM in a patient is analytically significant if it is higher than the reference change value (RCV), which is determined by the CV_a_ and CV_i_ through the mathematical expression:

$$\mathrm{RCV} (\%) =\sqrt{2} x Z x \sqrt{{\mathrm{CV}_{{i}}^{2}}+{\mathrm{CV}_{{a}}^{2}}}.$$

$$Z = 1.96\, \mathrm{for\,} \mathrm{a\,} \mathrm{confidence\,} \mathrm{level\, of\,} 95\%.$$

The theoretical basis of the RCV in relation to TMs is presented in a recent guideline from the SEQC^ML^ [8]. The TMs most used in breast cancer are carbohydrate 15.3 (CA15.3) and carcinoembryonic antigen (CEA). For CA15.3 and CEA, the CV_i_ is known (6.3–11.2% for CA15.3 and 9.9–12.9% for CEA), and the RCV can be calculated: 19–34% for CA15.3 and 30–40% for CEA [8]. Therefore, as a definition of significant analytical change, an increase > 25%, or more conservatively > 50%, over the previous concentration can be generally established. Some patients may have a base disease associated with a higher concentration and CV_i_, so the interpretation of the change in these populations requires knowledge of their initial CV_i_, which should be communicated by the laboratory involved.

A significant analytical change is not necessarily equivalent to a clinically relevant change. The interpretation, in addition to the consideration of the clinical context, is a function of the magnitude of the increase and the time between measurements, so it will depend on the growth rate of the tumour.

**Fig. 1:**
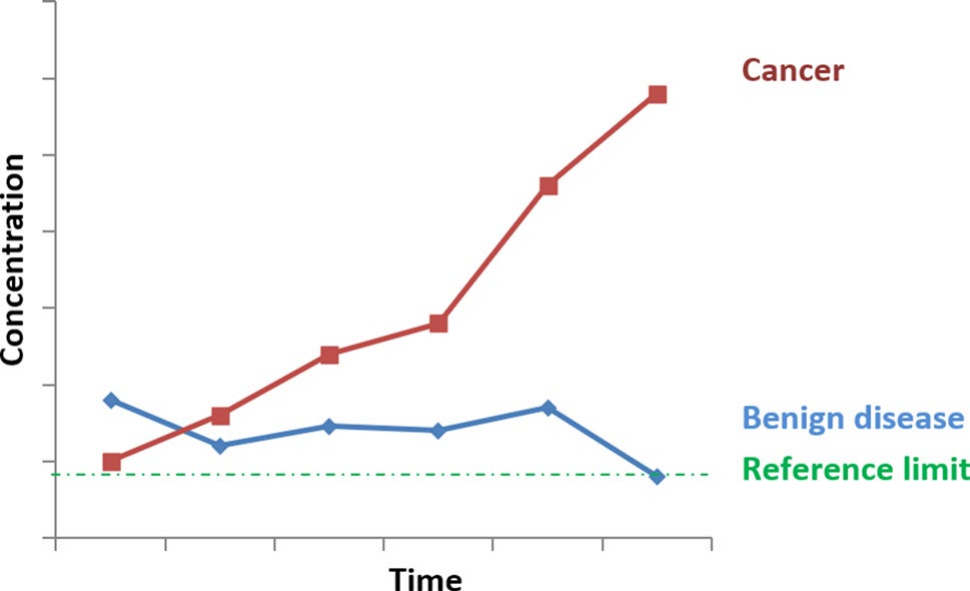
Example of changes in the levels of a tumour marker in the case of a benign (blue line) or malignant tumour (red line)

It is therefore necessary to correctly interpret any increase in a TM. In case there is a discrepancy between the clinical and analytical results, the following steps are recommended [7–9]: (i) ruling out a benign pathology (see below); (ii) investigating any methodological issues as mentioned above; and (iii) performing a second measurement 3–4 weeks later (or at least a period longer than the TM’s plasma half-life, which is 15–20 days for most); then evaluating the increase: (i) if the increase in concentration is < 15%, or if there is a decrease, then the change is not related to the evolution of the neoplasia and may be due to other reasons such as the analytical method or fluctuations in the TM itself; (ii) if the increase is 15–25%, then it is advisable to perform a second measurement 3–4 weeks (or as described above) later; (iii) if there are thus two separate increases > 25% or a single-period increase > 50%, then disease progression must be suspected. Therefore, the definition of a clinically significant change that marks the progression of neoplasia can be generally established as two separate increases of > 25% or a single-period increase > 50%, in the absence of benign pathology or methodological issues to explain it. This definition, not addressed in the American Society of Clinical Oncology (ASCO) or European Society for Medical Oncology (ESMO) guidelines, is in accordance with the guidelines of the European Group on Tumour Markers (EGTM) [9]. Performing serial determinations increases the specificity of the TM and allows changes unrelated to the tumour process to be detected. This is because in patients in whom benign disease is responsible for the increase in TM, variations usually occur in a “saw-toothed” fashion, while in those where the increase is due to disease progression, the increase is constant (Fig. 1).

In breast cancer, the most commonly used TMs are mucins, especially CA15.3 (although there are others, such as MCA or BR27.29), and CEA [9]. Other oncoproteins, such as human epidermal growth factor receptor 2 (HER2)/neu, can be used in HER2-positive breast cancer [10].

### TMs of highest value in breast cancer

CA15.3 is the soluble part of mucin-1, a high molecular weight protein that is located in the epithelium of ducts and mammary alveoli. Usually, the serum reference limit is 35 U/ml, depending on the laboratory and method of detection, and it is the TM of choice in breast cancer: its concentration rises in 20–50% of breast cancers [11]. In the detection of relapses, a sensitivity of 64% and a specificity of 99% have been described [11]. False positives of CA15.3 have been described in various situations (Table 1). Among them, elevations of up to 10 times in vitamin B12 deficiency and macrocytosis have been noted. Slight elevations have also been described in systemic inflammatory processes such as pneumonitis, myopathies, and autoimmune diseases, as well as with the use of granulocyte colony-stimulating factors (G-CSF) [12].

**Table 1:** Alternative causes of increased levels of TM in the absence of neoplasia [12]

Cause	CA15.3	CEA	HER2/neu
Vitamin B12 deficiency	+ + +		
Endometriosis	+	+	
Autoimmune disease	+	+	
Gastrointestinal disease	+	+	
Interstitial lung disease or pneumonitis	+ + +	+ +	
Hepatopathies	+	+	+
Hyperthyroidism	+		
Hypothyroidism		+	
Kidney failure	+	+	+
Inflammatory myopathies	+		
Treatment with G-CSF	+		
Smoking		+	

*CA15.3* carbohydrate antigen 15.3, *CEA* carcinoembryonic antigen, *G-CSF* granulocyte colony-stimulating factor, *TM* tumour marker

^+^Elevation up to 3 times the upper limit of the reference interval; ^++^elevation 3 to 10 times the upper limit of the reference interval; ^+++^elevation > 10 times the upper limit of the reference interval

CEA is a high molecular weight glycoprotein. Initially described in patients with metastasis of colorectal carcinoma, it is the most used TM in clinical practice and may be elevated in colorectal, gastrointestinal, pulmonary and breast cancers. It is encoded by 10 genes located on chromosome 19, whose physiological function is unknown but is probably related to mechanisms of cell recognition and adhesion [13]. Most of the healthy population have serum concentrations below 5 ng/ml, depending on the method of detection and the laboratory, although small increases (< 15 ng/ml) can occur in up to 5–10% of smokers. It is a nonspecific TM that is elevated in noncancer conditions such as liver disease, kidney failure, lung disease and hypothyroidism (Table 1) [12]. The joint determination of CA15.3 and CEA, depending on the cut-off point, has a sensitivity of 64–71% and a specificity of 86–99% for the detection of breast cancer relapse [9, 11, 14].

### Other TMs of potential value

HER2/neu, also called ErbB2, is a membrane protein with tyrosine kinase activity. This gene is overexpressed in HER2-positive breast cancer [15]. Its extracellular portion of 97–115 kDa can be measured in the serum. A normal level is usually below 15 ng/ml, depending on the method of detection and the laboratory. Although it can be expressed in other tumours, it has a high specificity in breast cancer, with discrete elevations also observed in cases of kidney and liver failure [12].

## Clinical utility of circulating TMs in breast cancer

Below, we present the clinical utility of the most relevant TMs in breast cancer for the diagnosis, follow-up and monitoring of the disease.

### Diagnosis

#### Early diagnosis or screening of a healthy population

None of the serum TMs known so far are sensitive enough to be used for breast cancer screening. For several years, the ASCO and the International Society of Oncology and Biomarkers (ISOBM) have not recommended the use of CA15.3 or CEA for breast cancer screening. Serum levels of these TMs below the reference limits in patients with suspected breast cancer do not exclude the presence of malignancy [9, 16].

#### Initial diagnosis of the disease

TMs are also not useful for the diagnosis of early-stage breast cancer, but they may be valid for the detection of advanced-stage breast cancer or metastatic disease. In 2005, the ISOBM EGTM recommended the serum measurement of CA15.3 and CEA as a useful complementary test to stage patients with breast cancer. Very high serum CA15.3 (> 50 U/ml) or CEA (> 20 ng/ml) in patients with breast cancer indicates metastatic disease. The serum concentration of CEA is elevated in 40–50% of patients with metastatic breast cancer, and the concentration of CA15.3 is elevated in 50–70% of cases. The evaluation of both TMs allows the diagnosis of metastasis, mainly in the bone and liver, in 60–80% of patients with breast cancer [9]. Preoperative serum levels of these TMs are correlated with the pathological stage of the tumour, depending directly on the size of the primary tumour and the presence of metastasis [17]. In the meta-analysis of Fu et al*.* [18], a total of 13 studies with 1179 breast cancer patients and 493 controls were analysed, and tumour stage was associated with serum levels of CA15.3 and CEA, which increased in concentration in patients with metastatic breast cancer. In another recent meta-analysis [5], Li et al. analysed 31 studies that evaluated CA15.3 levels and 23 studies that evaluated CEA levels in relation to breast cancer, in 12,993 patients overall. Subgroup analysis showed that elevated serum CA15.3 and CEA predicted the appearance of metastatic breast cancer. Elevated serum CA15.3 was associated with metastatic disease in younger women, while elevated serum CEA was associated with metastatic breast cancer in older women [5].

Thus, although the 2007 ASCO and the 2019 ESMO guidelines on breast cancer do not recommend the use of serum measurements of CA15.3 or CEA for the screening, diagnosis or staging of breast cancer [16, 19], it can nevertheless be concluded that published studies support an association between the existence of metastatic disease and elevated serum levels of CA15.3 and CEA in patients with breast cancer. Although none of the known TMs are useful for screening or early diagnosis of breast cancer, they may be useful for the diagnosis of metastatic disease in breast cancer. Although there is disagreement between the recommendations on the measurement of TMs for breast cancer staging by medical oncology societies (which give a negative recommendation) and societies of laboratory medicine (which give a positive recommendation) [9], elevated serum levels of CA15.3 or CEA in a patient with breast cancer have been associated with the presence of metastasis and such results should lead to more complementary tests aimed at confirming it [20].

### Early disease follow-up

In general, the main scientific societies (ASCO, National Comprehensive Cancer Network [NCCN], NACB, and ESMO) do not recommend measuring serum TMs for the monitoring of early breast cancer, given their low sensitivity and specificity [7, 16, 19, 21]. However, if levels are analysed before administering any therapy, they can be used as a reference value in subsequent evaluations, both in the follow-up of the patient and in the monitoring of responses to treatment.

In routine practice, some patients may have slightly elevated levels of a TM at the time of diagnosis and similar values after tumour excision, which would indicate that the TM levels were unrelated to the neoplasia. On the other hand, in patients without evidence of residual disease whose post-excision levels decrease, but without reaching the reference interval, the nadir is established for subsequent evaluations of the TM, which is of use because some comorbidities can lead to TM level elevation (Table 1).

Most commonly, however, TM levels are below the reference limit in early disease. In this case, once the initial treatments with curative intent in the early stages of the disease (surgery, chemotherapy, radiotherapy, hormonal therapy, and targeted therapy) are completed, the patient will undergo periodic evaluations to try to detect relapse. If TMs have been measured at baseline, serial serum level increases in asymptomatic patients may indicate the presence of micrometastases, which are not yet visible by imaging techniques nor produce symptoms. Even though TM elevations usually anticipate clinical and radiological evidence of relapse by 2–18 months, most clinical practice guidelines of oncological scientific societies do not recommend measuring CA15.3 or CEA during the follow-up of asymptomatic patients [7, 16, 19, 21]. This is due to the lack of evidence from prospective randomized trials demonstrating any impact on the survival or quality of life of these patients afforded by the early initiation of treatment [19]. In addition, the sensitivity of TMs in early diagnosis is related to the location of the recurrence, being lower in locoregional relapses and higher in patients with liver or bone metastases. However, the EGTM recommends the serial measurement of CA15.3 and CEA, at a frequency of 2–4 months for the first 5 years, biannually for the following 3 years, and annually thereafter [9].

Despite the recommendations to the contrary, in routine clinical practice, many health professionals request TM analysis during the follow-up of patients with asymptomatic early breast cancer. This is due to both the recommendations of the EGTM and the methodological limitations (statistical power and absence of risk stratification) of the studies that initially ruled out the benefit of early breast cancer follow-up with TMs, conducted more than two decades ago, when the availability of treatments that positively impacted survival was lower [22]. TM assessment should incorporate information about other conditions, both benign and pathological, that can alter their levels and could generate confusion in the interpretation of elevated results. This confusion can lead to unnecessary imaging tests that increase anxiety in patients. Therefore, as explained in Sect. 2.1, whenever there is a significant change in the level of a TM in patient follow-up, other conditions should be excluded, especially changes in liver and kidney function, that may explain its elevation (Table 1) [8, 23, 24]. When biochemical progression has been demonstrated with serial determinations of a TM, imaging techniques should be performed to confirm relapse. Radan et al*.* found recurrences on positron-emission tomography/computed axial tomography (PET-CAT) in 65% of patients with increases in CA15.3, CEA, CA125, and CA19.9 [25]. Di Gioia et al*.* obtained 66% sensitivity with CA15.3, CEA and CA125 in the detection of metastases, with 20% false positives [26]. Similarly, Göktas and Cayvarli observed a positive predictive value (PPV) for CA15.3 of 77% [27].

In patients with tissue overexpression of HER2/neu, the measurement of serum levels of the extracellular domain has also been proposed for the monitoring of the disease. Serial determinations can be useful for the early diagnosis of relapse, as it is a prognostic factor in disease-free survival and overall survival [28].

In summary, the clinical practice guidelines of ASCO and ESMO, among others, do not recommend serial measurements of levels of TMs for the early detection of recurrence in the follow-up of breast cancer, whilst the EGTM does recommend them. In cases in which a TM level was elevated at diagnosis, its subsequent measurement may be advisable to rule out any false positives, and serial measurements should be carried out to confirm the increases. In cases in which no TMs were elevated at diagnosis, the general recommendation is not to perform serial measurements of TMs for follow-up. If a centre elect to use such measurements for the early detection of relapse, the most appropriate strategy is the serial measurement of CA15.3 and CEA, which should probably be limited to high-risk cases. In the case of biochemical progression, regardless of whether a certain TM was elevated at diagnosis, it is advisable to carry out imaging tests to confirm relapse, which would lead to the initiation of treatment.

### In-treatment monitoring

The utility of serum TMs in the monitoring of breast cancer treatment is also controversial. Most studies and recommendations of scientific societies focus on mucins (CA15.3 and BR27.29) and to a lesser degree on CEA and serum HER2. TM measurement is recommended in combination with imaging tests, physical examination and symptom assessment, but not as the only means for monitoring response to treatment. However, in the absence of measurable disease, an increase in these markers may indicate progression while on oncological treatments. During the first 4–6 weeks of an oncological treatment, an increase in markers may be observed, which does not necessarily indicate a lack of efficacy. These types of increases are usually transitory [29, 30]. Other increases that are not associated with the progression of the disease are those observed in certain benign pathologies [2]. The definition of significant increase and marker progression (two consecutive increases of 25% or a single-period increase of 50%) has already been explained in the Introduction, and is the same in the context of metastatic disease [7, 31].

ASCO was the first association to publish clinical guidelines based on scientific evidence regarding the use of TMs in breast cancer, in 1996 (latest update in 2007), and it has not modified its recommendations in subsequent guidelines [29].

#### Neoadjuvant treatment

The role of TM monitoring in neoadjuvant treatment is controversial, as there are publications that correlate these marker levels with tumour response [32, 33], while in others this correlation is not observed [34, 35]. According to ASCO guidelines, there is insufficient evidence to recommend TM monitoring during neoadjuvant treatment.

#### Advanced disease

The monitoring of patients with advanced disease and the evaluation of response to treatment should be done not only by assessing the observed changes in levels of TMs but also with a consideration of relevant clinical information [16, 31, 36]. Only when the degree of disease involvement could not be quantified, a significant increase in levels of mucins (CA15.3 and BR27.29) or CEA in the absence of an increase in mucins according to the established progression criteria may indicate the ineffectiveness of the treatment and the need to consider discontinuing it [29]. This concept is supported, with small variations, by the main clinical practice guidelines. Thus, NCCN recommends, together with clinical and laboratory criteria, the evaluation of mucins and CEA in patients with advanced breast cancer [21]. According to ESMO guidelines, CA15.3 and BR27.29 can be useful to assess responses to treatment of patients with advanced disease, but only in specific situations such as when it is otherwise not possible to evaluate the response [37]. NACB mainly proposes the assessment of mucin levels (CA15.3 and BR27.29) as well as CEA, along with clinical examination and imaging tests, to evaluate responses to treatment of patients with advanced disease, in particular as ESMO guidelines suggest, in patients in whom the degree of involvement is not quantifiable (such as bone disease). In this case, the verification of the progression of TM levels suggests the progression of the disease, which raises the possibility of discontinuing or modifying treatment, or enrolling the patient in a clinical trial [7]. The EGTM advises the measurement of mucins (CA15.3 and BR27.29) and CEA before each chemotherapy cycle in patients with advanced breast cancer and at least every 3 months in patients receiving hormone therapy [9].

With respect to the choice of TM, two multicentre studies [33, 38] demonstrated that variations in CA15.3 correlated with response to treatment. The marker can be useful for monitoring especially if levels are elevated at the beginning, but caution must be exercised when considering changes in treatment, by ruling out non-tumour causes or non-significant elevations, as described above [39]. Although the evaluation of CEA is included in the different guidelines, its applicability is more controversial, and it has more limited utility than mucins (CA15.3 and BR27.29), especially in the assessment of patients when mucins do not increase. Regarding serum HER2, NACB alone considers it of value for assessing response to treatment but only in patients with advanced disease who are treated with trastuzumab [7, 40, 41].

The evidence from the different guidelines on the usefulness of TMs in the follow-up of metastatic breast cancer is level III at best and level II for monitoring response to adjuvant therapy [37]. These levels of evidence are supported by the opinions of expert groups based on their clinical experience and the results of descriptive studies conducted by several centres. Their degree of recommendation does not exceed grade C, and it is common to find contradictory conclusions on this topic, which makes it difficult to make recommendations for or against. As a general recommendation of this consensus document, and considering routine clinical practice and the recommendations of most clinical practice guidelines, the usefulness of TMs for treatment monitoring in patients with advanced breast cancer who have elevated baseline levels should be noted, especially when the disease is difficult to evaluate by imaging. Serial measurement is not recommended in patients with advanced disease who do not have elevated levels at the beginning of treatment.

## Recommendations

Table 2 shows the consensus recommendations for utilizing circulating TMs in patients with breast cancer.

**Table 2:** Consensus recommendations

1. For the measurement of levels of TMs in patients with breast cancer, the preanalytical aspects (adequacy of the sample) and analytical aspects (use of the same method, assessment of possible analytical issues, etc.) should be adequately addressed
2. In breast cancer, the recommended circulating TMs of highest value are CA15.3 and CEA. CA15.3 is the most strongly recommended marker; CEA is supported by less evidence. Serum HER2 has only potential use in advanced HER2-positive disease with trastuzumab-based treatments
3. In patients without underlying pathology but with elevated marker levels, an increase of at least 25% or, more conservative, of at least 50% over the previous levels detected is considered a significant analytical change
4. In cases where there is a discrepancy between the clinical evaluation and an elevated level of the marker, technical issues of the analysis should be investigated, benign pathology ruled out (vitamin B12 deficiency, gynaecological, gastrointestinal, pulmonary, autoimmune, thyroid, renal, or muscular), treatment effects ruled out, and a second measurement should be made 3–4 weeks later
5. Biochemical progression of the marker is considered to have occurred if there are two consecutive increases of at least 25% or a single-period increase > 50% in the absence of benign pathology or methodological issues that might otherwise explain it
6. To interpret the changes in TM levels in patients with other diseases associated with elevated levels or when there is discrepancy with the clinical evaluation, consultation with the laboratory physician is recommended
7. The measurement of circulating TMs is not recommended for screening or early diagnosis of breast cancer
8. In patients with early breast cancer in whom a TM is elevated at diagnosis, serial measurements are advised to rule out or confirm that the elevation is related to other causes
9. In patients with early breast cancer and normal TMs at diagnosis, serial TM measurements are not generally recommended for a follow-up aimed at detecting relapse due to the absence of a demonstrated impact on survival or quality of life. In those centres that elect to measure a TM, this should be limited to patients with high risk of relapse, and the most appropriate markers are the combination of CA15.3 and CEA
10. It is recommended to measure CA15.3 and CEA in patients with a suspected or confirmed diagnosis of metastatic breast cancer
11. The use of TMs to monitor the response to neoadjuvant breast cancer treatment is not recommended
12. Periodic measurement of TMs to monitor responses to treatment in patients with advanced breast cancer can be useful in combination with other clinical tests, especially when the disease is not measurable or easily evaluable by imaging studies, but is not recommended as a single tool. Before assessing the biochemical progression of TMs, imaging tests should be performed to confirm the relapse or progression of the disease

## Conclusions

The relevance of breast cancer as the leading neoplasia in women and the complexity of its treatment approach have motivated the writing of this consensus article, which aims to improve the use and interpretation of circulating TMs in patients with breast cancer. The text summarizes the current knowledge and available evidence on the subject, trying to bring closer the approach of clinical laboratory specialists and medical oncologists. The recommendations we have developed mainly focus on the indication, the frequency of measurements and the factors that must be taken into consideration for the correct interpretation of changes in TM levels. The goal of this effort, which is the result of a collaboration between the SEQC^ML^ and SEOM, is to facilitate the multidisciplinary work of professionals in centres where patients with breast cancer are cared for. Ideally, this consensus should lead to the establishment of joint working protocols that set the indications, testing frequency and interpretation criteria of circulating TMs in breast cancer in each centre. This is a preliminary work that should be followed up with consensus articles on other pathologies, which will bring together the two medical specialties that have been traditionally separate but whose collaboration is desirable in the future.
